# The effect of hypnosis on pain relief due to injection of dental infiltration anesthesia

**DOI:** 10.1002/cre2.356

**Published:** 2021-04-07

**Authors:** Soma Arabzade Moghadam, Fayegh Yousefi, Sahand Saad

**Affiliations:** ^1^ Pediatric Dentistry School of Dentistry Kurdistan University of Medical Sciences Sanandaj Iran; ^2^ Neurosciences Research Center Kurdistan University of Medical Sciences Sanandaj Iran; ^3^ Kurdistan University of Medical Sciences Sanandaj Iran

**Keywords:** anesthesia injection, behavioral control, dental anxiety, hypnosis, pain

## Abstract

**Background and objective:**

Dental patients often experience the fear of pain induced by injectable anesthesia. This study aimed to investigate the impact of hypnosis on relieving the pain of injected dental infiltration anesthesia.

**Materials and methods:**

This single‐blind clinical trial was conducted on 32 healthy volunteers to assess the pain perception in mucosal injection. The visual analog scale was applied for the measurement of one‐sided pain intensity in the maxilla without hypnosis. When hypnosis was implemented, the same procedure was performed on the other side of the maxilla reversely within one session.

**Results:**

Hypnosis implementation significantly decreased the intensity of the perceived pain before anesthesia injection (*p* = 0.05).

**Conclusion:**

Hypnosis before the injection of dental infiltration anesthesia could decrease the pain intensity caused by the injection. Therefore, hypnosis therapy is recommended as an effective approach to pain control for anesthesia injection.

## 1 INTRODUCTION

Today, dentistry practice has been facilitated by technological and material advancement and trailblazing infection control. Local anesthesia injection is speculated to be a stimuli that causes anxiety in dental patients (Shapiro et al., 2002), and needle phobia may adversely affect some of these patients (McDonnell‐Boudra et al., 2014). The effective management of the anxiety and pain induced by any form of injection is paramount; improper measures in this regard lead to increased pain and discomfort following injection. One the consequences is needle phobia, which discourages effective medical prevention and diagnosis (Taddio et al., 2010).

In general, pain and anxiety are experienced together (Appleton et al., 2010), and meticulous dentistry programs must be considered for the patients with an uncontrollable fear of dental procedures. Anesthesia injection may cause pain, the intensity of which is affected by factors such as the needle gauge, anesthesia type and temperature, and injection site pH (Kaufman et al., 2005).

Stress management could be accomplished by numerous medicinal and non‐medicinal techniques, an important example of which is hypnosis (Armfield & Heaton, 2013; Glaesmer et al., 2015; Kekecs et al., 2016). Anxiety is an emotional state for protection against various perceived threats. Dental anxiety is the specific response of patients to the stress induced by dental health (Glaesmer et al., 2015). According to statistics, one‐seventh of the population are extremely anxious about dental treatments, and proper measures are expected on behalf of dentists in the case of these patients; pharmacological and non‐pharmacological (behavioral and cognitive) behavioral management techniques have been shown to be effective in this regard (Armfield & Heaton, 2013).

Perpetual human research has brought about substantial changes in various fields of science, including psychology. Hypnotherapy is an important aspect of psychology, which has proven beneficial in human psychological and psychological treatments, which has been explored and advanced by overcoming the initial challenges. Clinical dental practices have also been positively influenced by the technological and material advancement in this regard. Nevertheless, the pain, anxiety, and negativity associated with undergoing dental procedures continue to affect the patients, causing significant dental care challenges worldwide (Nigam et al., 2013).

Hypnosis has proven effective in solving the medical and psychiatric issues of various patients. Hypnosis refers to a psychological state involving focused attention to utterly decrease perceived environmental awareness (Trakyali et al., 2008), acting as a remarkable pain and anxiety relief measure in different patients (Kekecs et al., 2016).

Anxiety covers a wide range of phenomena in many populations and is not merely focused on a specific issue. Anxiety may give rise to various phobias, such as the fear of dental treatment. As a variable, pain cannot be evaluated objectively due to the varying unpleasant sensations in patients cognitively, emotionally, and socially (Uman et al., 2013). The combination of pain and anxiety also cause challenges in pediatric dental procedures, especially with anesthesia (Ramírez‐Carrasco et al., 2017).

The pain threshold of patients could increase by psychological interventions, which also minimize the complications associated with vaccine injections (e.g., pain and the consequences). As a tested psychological intervention, hypnosis effectually decreases the pain and discomfort caused by needles in dental patients (Birnie et al., 2014; Birnie et al., 2018). Similar interventions have also been reported to diminish excessive needle phobia (Birnie et al., 2015). Recent findings have indicated that hypnotherapy significantly decreases pain in dental patients.

This research aimed to determine the impact of hypnosis on relieving the pain induced by the injection of dental infiltration anesthesia and compare the mean difference in the pain intensity between the case and control groups.

## 2 MATERIALS AND METHODS

This single‐blind clinical trial was conducted on 32 randomly selected volunteers aged 18–25 years who underwent restorative dentistry of the anterior maxilla, which was symmetrical in the left and right maxilla. The induction of hypnosis was performed in the form of mucobuccal fold pretreatment during injection above the maxillary first premolar. The research objectives were explained to the participants, and written informed consent was obtained prior to enrollment.

Based on the propositions of Huet et al. (2011), the mean values of 1.07 ± 1.05 and 2.16 ± 2.80 were considered for the case and control groups, respectively at 95% confidence interval and 80% test power, and the sample size formula was as follows:

$$n={\left(Z_{1}-\alpha /2+Z_{1}-\beta \right)}^{2}\left({S^{2}}_{1+}\;{S_{2}}^{2}\right)/{\left(\mu_{1-}\;\mu_{2}\right)}^{2}$$

The minimum sample size for each group was 16, while the minimum total sample size was 32.

**FIGURE 1:**
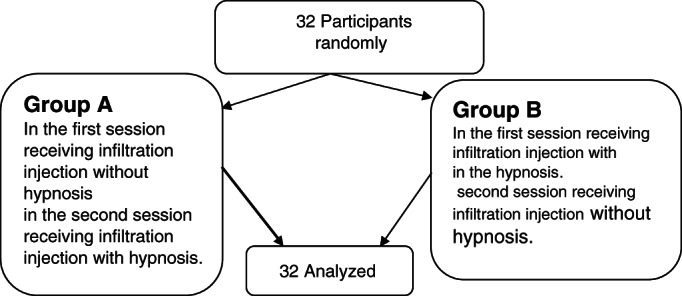
Flowchart of sample selection

After the selection of the participants by random sampling, they were allocated to the case and control groups via quadruple blocks. In total, 32 patients were randomly assigned to two groups (*n* = 16) of A, who underwent infiltration injection in the first session without hypnosis (hypnosis in the second session, followed by injection), and B, who underwent hypnosis in the first session, followed by injection (infiltration injection in the second session without hypnosis) (Figure 1). Notably, the A and B classification of the study groups differed with the classification of the subjects as the case and control groups as the control group included the patients whose jaw received no hypnosis, and the subjects underwent injective anesthesia, while the intervention group referred to the patients undergoing injective anesthesia following hypnosis.

The participants aged >18 years, requiring the double‐sided restoration of the first premolar teeth in the jaw, and willing to undergo hypnosis were considered eligible for enrollment. The researchers carefully addressed the concerns of the subjects regarding inadequate knowledge of hypnosis, as well as their possible misconceptions. After informed consent was obtained in the written form, the systemic status of the subjects was examined using a designated form for this purpose. However, the perioperative anxiety status of the patients was not evaluated since the parameter was unrelated to the study objectives. No complications were caused by the intervention.

As recommended by the American Society of Anesthesiologists (ASA), treatment contraindications (e.g., cardiac disorders, untreated epilepsy, severe psychiatric disorders) and low IQ (diagnosed by a psychologist based on clinical suspicion) were the exclusion criteria of the present study. In the ASA classification, ASA I refers to healthy status, and ASA II is indicative of mild‐to‐moderate systemic diseases due to surgeries or other pathological processes that are properly controlled medically. Our patients fell under the ASA I category (Knuf et al., 2018).

After the confirmation of the patients' status, hypnotherapy was implemented to assess the suggestibility of the patients by locking hands, which is a common approach to hypnosis. If the patients were unable to concentrate or accept hypnosis, they would be excluded, and in case of a positive test response, they would be enrolled.

### 2.1 Research tools

#### 2.1.1 Materials and instruments

The injections were performed using a short needle (25 mm), gauge 27, and 2% lidocaine (xylocaine) for anesthesia; notably, lidocaine contained epinephrine (1:80,000). The depth of the needle point, direction of the needle bevel, temperature of the carpool fluid, and speed of the anesthetic injection were the same.

#### 2.1.2 Data recording

After the injection, pain relief was measured using the visual analog scale (VAS) (Aitken, 1969), which is composed of a horizontal line (length: 10 cm) marked with the phrases “No Pain” on the left and “The Worst Pain Possible” on the right. The respondents mark the level of their perceived pain on the linear scale, knowing that the beginning and end of the scale indicate the lack of pain and severe pain, respectively. To do so, the respondent places a hand on the spot corresponding to their pain intensity, selecting a number within the range of 1–10 to indicate the intensity of the perceived pain. In this study, an individual blinded to the data of the subjects recorded the data at this stage.

#### 2.1.3 Procedures

Patient selection was based on the inclusion and exclusion criteria, and the number of the treatment sessions was determined in accordance with the protocol. The potential benefits and drawbacks of the research project were fully explained to the subjects as the project involved human models. The subjects who were willing to participants provided their written informed consent and were allowed to withdraw from the study at any given time.

Hypnosis was carried out in one treatment session to decrease the patients' pain using the indirect hypnosis techniques, pain transfer, dissociate, telescope, and distraction techniques through several stages, such as the preparation of the patients for hypnosis, hypnosis, deepening techniques, therapeutic hypnosis, and termination. We applied the hypnosis technique proposed by Erickson as appropriate for dental patients (Goldenberg & Goldenberg, 2012). In the deepening stage, we employed techniques such as staircase visualization and counting, and injective local anesthesia was implemented afterwards onto the patients' hands. The concentrations of epinephrine and injectable lidocaine were completely carpooled for each side of the maxilla (1.8 cc of 2% anesthetic lidocaine solution, 0.18 of epinephrine).

After the mentioned procedures, self‐report data were collected from the patients regarding the numbness of their hands (responding in the trance mode by pointing the finger of the other hand). The injected anesthesia targeted the desired region of the teeth, so that the patients could touch the tooth and soft tissue with their finger. Following that, dental infiltration anesthesia was injected to the region requiring restoration (i.e., maxillary premolar on either side), and appropriate post‐hypnosis empathy was also provided, in addition to the previous presentations of the hand and tooth anesthesia removal. The restoration continued until reaching the state of pre‐hypnosis. In another session on the symmetrical side of the jaw, injection was performed without hypnosis. Notably, the selection of the patients and sides to receive injection with or without hypnosis was completely random.

#### 2.1.4 Statistical analysis

SPSS version 20 was used for data analysis, and the demographic variables were expressed as mean, standard deviation, frequency, and percentage. Independent *t*‐test was also applied to address the analytical objectives of the research considering the significance level of 0.05.

#### 2.1.5 Ethical considerations

The Ethics Committee of Kurdistan University of Medical Sciences, Iran approved the protocol of this research project (IRB No. REC.IR.MUK 1397/18). The patients had a companion during all the stages in the medical department, and a clinical psychologist performed the hypnosis session, a dentist was responsible for the injections and treatments, a supervisor (pediatric dentistry) was in charge of data recording, and a statistical consultant performed the data analysis. This study has been registered in the Iranian Registry for Clinical Trials (IRCT20141756401756N3; http://irct.ir/).

## 3 RESULTS

**FIGURE 2:**
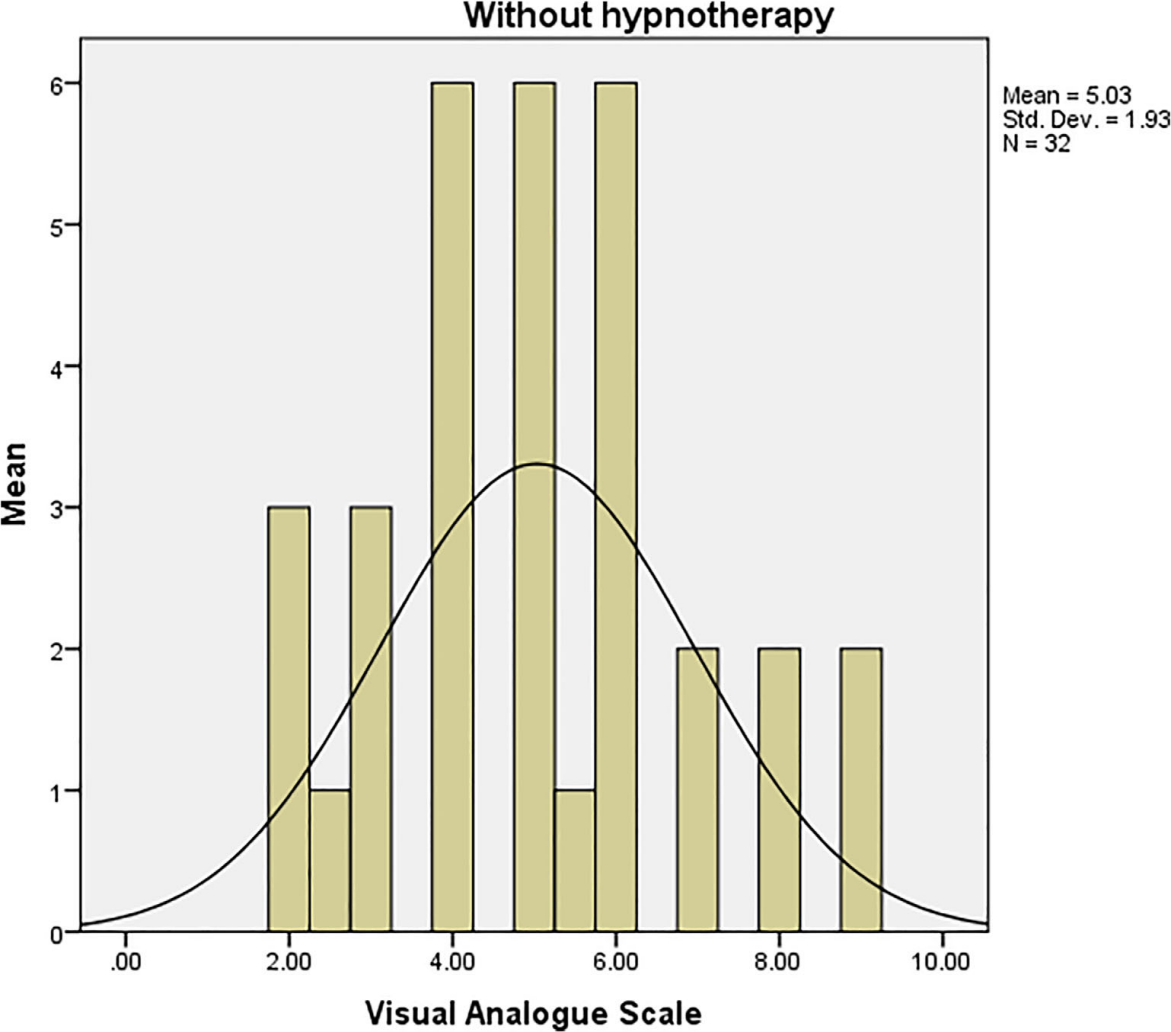
Mean pain control without hypnotherapy based on VAS

**FIGURE 3:**
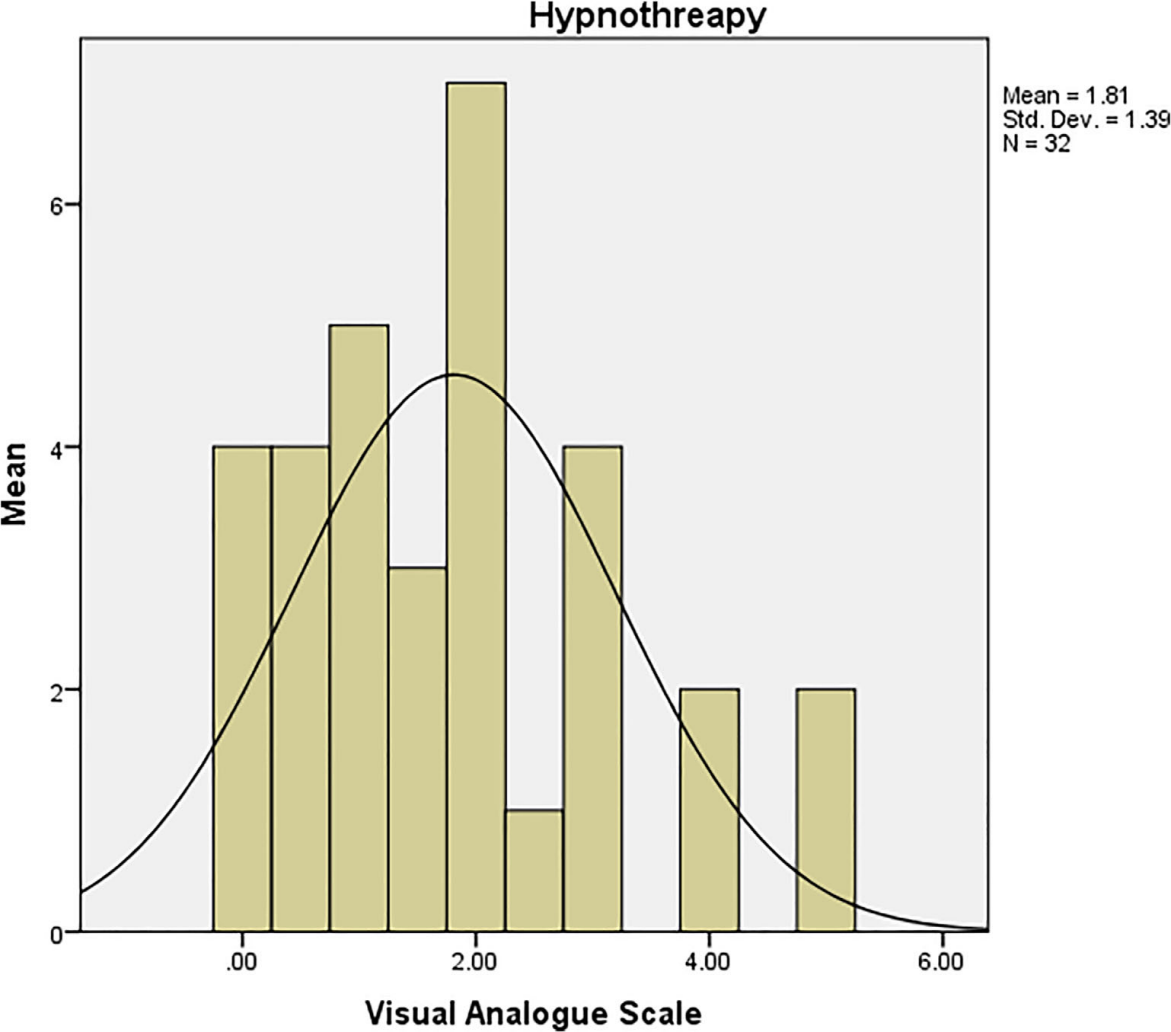
Mean pain control with hypnotherapy based on VAS

The frequency distribution of the demographic variables (esp. gender) of the subjects is presented in Table 1. In total, 16 women and 16 men were enrolled in the study (43.8% and 56.3% of men in groups A and B, respectively and 56.3% and 43.8% of women allocated to groups A and B, respectively). The findings in Table 1 indicated no significant correlations between the demographic variables of the subjects in the groups (*p* = 0.0.5). Based on the VAS, the mean pain control with and without hypnotherapy was 1.81 ± 1.39 and 5.03 ± 1.93, respectively (Figures 2 and 3). According to the information in Table 1, three subjects in group A (60%) and two subjects in group B (40%) were employed, while 14 subjects in group A (50%) and 14 subjects in group B (50%) were unemployed.

**TABLE 1:** Injection with and without hypnotherapy based on demographic variables

Variables	Group A^a^	Group B^b^	*p*‐Value
	*N* (%)	*N* (%)	
Gender			0.48
Male	7 (43.8)	9 (56.3)	
Female	9 (56.3)	7 (43.8)	
Age (*M* ± SD)	23.93 ± 1.84	23.56 ± 1.82	0.56
Education level			0.37
High school	12 (54.54)	10 (45.45)	
University	4 (40)	6 (60)	
Occupation status			0.0.5
Employed	3 (60)	2 (40)	
Unemployed	14 (50)	14 (50)	

a Injection of numbness without hypnotherapy.

b Injection of numbness with hypnotherapy.

According to the findings, groups A and B were significantly different regarding the injected anesthesia with and without hypnotherapy (*t* = −2.12; *p* = 0.04) (Table 2).

**TABLE 2:** Mean differences between two groups include injection of numbness without hypnotherapy and injection of numbness with hypnotherapy

Groups	*n*	*M* ± SD^a^	df	*t*	*p*
			30	2.12	.04
Injection of numbness without hypnotherapy	16	4.34 ± 1.32			
Injection of numbness with hypnotherapy	16	5.71 ± 2.22			

a Mean and standard deviation.

## 4 DISCUSSION

In the current research, the impact of hypnotherapy on the pain intensity induced by injecting dental infiltration anesthesia was investigated, and the mean pain intensity after the injective anesthesia without hypnosis was estimated at 5.03, which is in line with the findings of Armfield et al. According to the results of the present study, the mean perceived pain without hypnosis in groups A and B was 4.34 and 5.71, respectively, while the intensity decreased to 1.50 and 2.12, respectively after the intervention with a significant difference in this regard (*p* = 0.05). This is consistent with the previous studies in this regard, which have demonstrated that pain intensity based on the VAS could significantly reduce from 7.1 to 4 following hypnotherapy as also confirmed in another investigation (Arons, 2014; Wolf et al., 2016).

A similar research (Abdeshahi et al., 2013) was focused on the impact of hypnosis on local anesthetic injection and perceived pain during the extraction of the third molar using hypnosis. According to the findings, half of the patients in the hypnotherapy group required postoperative analgesics, which confirms the effectiveness of hypnosis in pain relief in dentistry and in in congruence with our findings. The current literature shows the positive, significant impact of hypnosis on pain relief in dental procedures in line with the findings of the current research (Abdeshahi et al., 2013; Bidar et al., 2009; M. S. evaluation, 2017; Glaesmer et al., 2015; Huet et al., 2011; Kekecs et al., 2016).

Hypnosis effectually relieves the pain and anxiety caused by injection in dental patients (Birnie et al., 2014). According to the study by Bidar et al. (2009), hypnosis and local anesthesia in root canal treatment was effective in 76.2% of the patients, while unsuccessful in 23.8%, and the difference in the success rate was significant. Hypnosis is a viable therapeutic option for anesthetists, surgeons, and dentists (Hermes et al., 2004) and is also recommended as an alternative to various therapies (Jensen et al., 2010). The effects of hypnotherapy in various fields and medicine on pain relief have been widely explored, and recent findings have also shown the effectiveness of self‐hypnosis training in relieving headache and decreasing pain intensity, duration, and frequency per week (Kohen & Zajac, 2007). Furthermore, the results obtained by Schwebel et al. (2002) regarding the influence of hypnotherapy on chest pain of non‐cardiac origin demonstrated that after 12 sessions, pain intensity and medicine intake significantly declined (Schwebel et al., 2002). Similarly, the findings of Tan et al. (2015) were indicative of the significant reduction in the pain intensity of the patients receiving hypnosis. In another research, hypnosis was found to be highly effectively in the pain control of the pediatric patients with cancer and chronic pain (Tomé‐Pires & Miró, 2012).

In an investigation in this regard, hypnotherapy could significantly decrease the anxiety of the patients admitted to a private clinic in Tehran (Iran) by 43.3% in the experimental group after hypnosis (Lotfifar et al., 2013). Mirzamani (2012) has also reported the effectiveness of hypnotherapy in disease treatment.

Our findings were indicative of no significant correlations between pain relief after hypnosis and the variables of age, gender, and education level. According to the literature, hypnosis tends to affect women more significantly compared to men (Yeates, 2016), and the success rate could be attributed to the willingness of women for a psychological state for therapeutic purposes (Yeates, 2016). Due to the error in data analysis and impact of the small sample size of the present study, the significance or insignificance could not be determined accurately in terms of the occupation status.

Individuals with severe anxiety have a low threshold for pain, which exposes them to high levels of psychological stress in dental procedures. Hypnotherapy increases the pain threshold and reduces the pain induced by local anesthesia in these patients, and the pain tolerance improves in the case of local anesthesia (Ghadimi Gili et al., 2016). At dental clinics, patients clearly have high stress levels and a low threshold for pain. In hypnotherapy, determining the cause of stress and taking the necessary measures enhances the interactions between the patients and therapists, which in turn leads to the higher possibility of the permanent resolution of the clinical condition. In some cases, an empathic relationship between the patient and therapist could act as a straightforward solution to the anxiety of the patient (Mehrani & Poorasghar, 2016).

Hypnotherapy is highly beneficial in disease treatment and remarkably decreases anxiety if implemented by skilled dentists and specialists (Roberts, 2006). Hypnosis refers to an altered state of consciousness linked with high hypnosis suggestibility, which contributes to pain relief in various patients through diminishing pain perception. Moreover, the induction of a positive illusion by hypnosis increases cognition through imagining the affected region, thereby alleviating the perceived pain (Ghadimi Gili et al., 2016).

Although neural mechanisms remain unclear, recent findings have proposed the key role of the femur bone and primary sensory cortex in sensory pain perception (Berger et al., 2010). As such, the impact and pain relief would be palpable through reduced anxiety. Pain is a complicated phenomenon and quite difficult to manage by any particular techniques (Dillworth & Jensen, 2010).

This was the first investigation in this regard in Iran. One of the limitations of the research was that some of the patients were afraid of hypnosis, which was resolved by providing the necessary information by a clinical psychologist. Considering the subject matter (i.e., hypnosis), another limitation was the small sample size as many patients were unwilling to partake in the intervention. Finally, there might have been bias affecting the obtained results, which was resolved by the random selection of the participants.

## 5 CONCLUSION

Hypnosis could effectively decrease the perceived pain induced by dentistry injection into the maxillary buccal mucosa, and hypnotherapy is recommended as a reliable and beneficial measure in dentistry. Notably, the success rate of hypnotherapy largely depends on the hypnosis level of the patients.

## CONFLICT OF INTEREST

None declared.

## Data Availability

The data that support the findings of this study are available from the corresponding author upon reasonable request.
